# Assessing the Population Demographic History of the Tsushima Leopard Cat and Its Genetic Divergence Time from Continental Populations

**DOI:** 10.3390/biology14070880

**Published:** 2025-07-18

**Authors:** Hideyuki Ito, Nobuyoshi Nakajima, Manabu Onuma, Takushi Kishida, Miho Inoue-Murayama

**Affiliations:** 1 Kyoto City Zoo, Kyoto 606-8333, Japan; 2 Biodiversity Division, National Institute for Environmental Studies, Tsukuba 305-8506, Japan; 3 Museum of Natural and Environmental History, Shizuoka 422-8017, Japan; 4 College of Bioresource Sciences, Nihon University, Fujisawa 252-0880, Japan; 5 Wildlife Research Center, Kyoto University, Kyoto 606-8203, Japan

**Keywords:** PSMC, SMC++, leopard cat, effective population size, genetic diversity

## Abstract

The Tsushima leopard cat (*Prionailurus bengalensis euptilurus*), an endangered feline endemic to Tsushima Island, Japan, faces critical threats due to its small and isolated population. Understanding its demographic history and genetic differentiation from continental populations is essential for conservation planning. In this study, we performed whole-genome sequencing of four Tsushima individuals and applied demographic inference methods, including pairwise sequentially Markovian coalescent (PSMC) and Sequentially Markovian Coalescent (SMC++), to reconstruct the historical effective population size (*Ne*) and estimate divergence times. PSMC revealed a population expansion between 200,000 and 100,000 years ago, followed by a long-term decline. SMC++ inferred a continuous decline and estimated that the divergence from the Korean leopard cat population occurred approximately 30,000–20,000 years ago. Genetic diversity analysis showed that the Tsushima population has significantly lower heterozygosity and higher inbreeding levels than continental populations. Genetic clustering based on genome-wide SNPs indicated that the Tsushima population is genetically closest to the Korean population, forming a northern cluster distinct from southern populations, such as Borneo and the Malay Peninsula. These findings provide valuable insights into the evolutionary history and genetic status of the Tsushima leopard cat and contribute critical data for the design of future conservation strategies targeting this unique insular lineage.

## Simple Summary

The Tsushima leopard cat is a small wild feline that lives only on Tsushima Island in Japan. Its population is critically endangered, with around 100 individuals remaining in the wild. Understanding its genetic background is essential for effective conservation. In this study, we analyzed the entire genome of several Tsushima leopard cats and compared them with leopard cat populations from other regions of Asia. We used advanced computational methods to estimate how their population size has changed over time and when they became genetically separated from mainland populations, such as those in Korea. Our results show that the Tsushima population has been shrinking for thousands of years and became isolated from the Korean population around 30,000–20,000 years ago. We also found that the Tsushima leopard cat has low genetic diversity and signs of inbreeding, which may reduce its ability to adapt to environmental changes. These findings highlight the need for urgent conservation action and suggest that genetic monitoring should play a key role in preserving this unique and vulnerable population. Our study provides valuable insights into the species’ history and supports future planning for its protection.

## 1. Introduction

**Figure 1:**
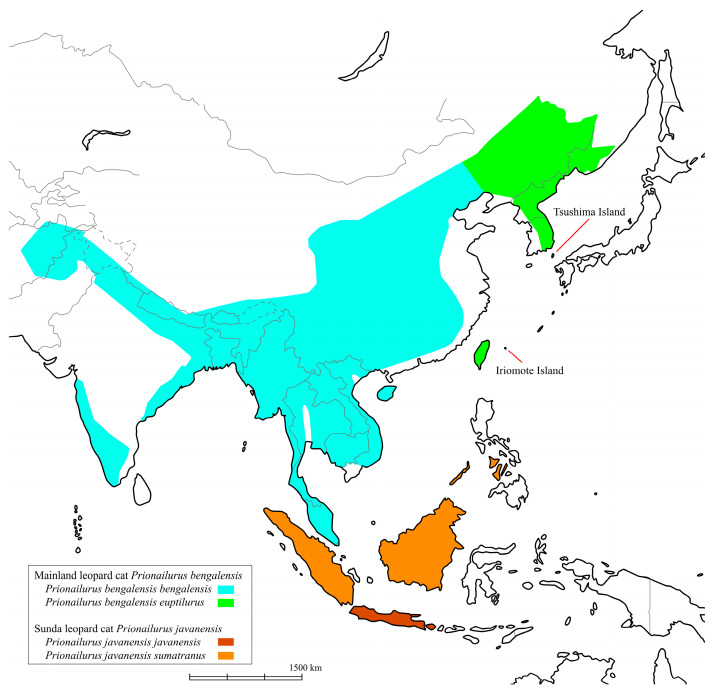
Distribution of the two leopard cats: mainland leopard cats (*Prionailurus bengalensis*) and Sunda leopard cat (*Prionailurus javanensis*). The classification is based on [3].

The leopard cat (*Prionailurus bengalensis*) is widely distributed in Asia—from as far west as Pakistan and Afghanistan, through parts of India and the Himalayan foothills, to most of China, the Korean Peninsula, the Russian Far East in the north, and as far south as Sumatra, Java, Borneo, and Taiwan (Figure 1) [1]. It is classified into 12 subspecies according to Wozencraft’s classification [2]. In another classification, the northern populations, such as those in China, eastern Russia, and Japan, are classified as mainland leopard cats (*Prionailurus bengalensis*), while the southern populations, such as those in Borneo and Sumatra, are considered a separate species, the Sunda leopard cat (*Prionailurus javanensis*) [3]. While many leopard cat populations remain relatively stable and are categorized as Least Concern on the IUCN Red List, declines have been reported in some regions, such as Japan and Korea, where they are designated as protected species [1]. In Japan, the Tsushima leopard cat (TLC) and the Iriomote leopard cat (ILC) inhabit the Tsushima and Iriomote islands, respectively (Figure 1). By Wozencraft’s classification [2], the TLC is identified as a regional population of the Amur leopard cat (*Prionailurus bengalensis euptilurus*) while the ILC is identified as a different subspecies (*Prionailurus bengalensis iriomotensis*). By contrast, Kitchener et al. [3] classified both these cat populations as subspecies of the mainland Amur leopard cat. Both TLC and ILC populations are estimated at approximately 100 individuals each and are listed as threatened species by the Japanese Ministry of the Environment. Various conservation efforts are underway. This taxonomic distinction is critical for conservation and management. Moreover, understanding how populations have been established can help define appropriate conservation units.

Given this background, assessing the genetic diversity and demographic history of endangered species is fundamental to understanding their evolutionary potential and informing effective conservation strategies [4]. Genetic variation within and among populations underlies their capacity to adapt to environmental changes and is therefore a cornerstone of long-term species persistence. Furthermore, clarifying phylogenetic relationships and population genetic structure enables the identification of evolutionarily significant units (ESUs), which are critical for prioritizing conservation efforts. Demographic inference can also reveal historical events—such as population bottlenecks, expansions, or periods of isolation—that have shaped contemporary genetic patterns. These insights help anticipate future vulnerabilities and guide the development of targeted management interventions, such as genetic rescue, translocation, and captive breeding programs. Effective population size (*Ne*)—the number of individuals contributing to genetic diversity—is a fundamental parameter in population genetics. Although *Ne* is often lower than the actual census size, it allows us to consider both the number of individuals and the genetic diversity. The pairwise sequentially Markovian coalescent (PSMC) and sequentially Markovian coalescent (SMC++) can be used to estimate the changes in effective population size over the past several million years using whole-genome data from one or multiple individuals, respectively [5,6]. The PSMC and SMC++ are also useful for estimating the time of divergence between populations.

The aim of this study was to estimate the demographic history of the TLC and its divergence time from the continental populations using whole-genome sequencing.

## 2. Materials and Methods

### 2.1. Samples

This study strictly adhered to the ethical guidelines for animal research established by the Wildlife Research Center of Kyoto University. The research protocol was approved by Kyoto City Zoo and the Ministry of the Environment (Approval No. #20170426; Kyoto City Zoo Ethics Committee). We obtained muscle samples from four wild TLC individuals and a cultured cell line derived from a wild ILC individual. The muscle tissues were preserved in ethanol until DNA extraction, which was performed using the DNeasy Blood and Tissue Kit (QIAGEN, Hilden, Germany). DNA concentrations were measured using both a NanoDrop spectrophotometer and a Qubit fluorometer (Thermo Fisher Scientific, San Diego, CA, USA).

### 2.2. Sequencing

The isolated genomic DNA was used to construct short-insert libraries using TruSeq DNA PCR Free (350) (Illumina, Cambridge, MA, USA), following the manufacturer’s protocols. Subsequently, 150 bp paired-end sequencing was performed using a whole-genome shotgun strategy on either the Illumina HiSeq X Ten or NovaSeq platform. Library preparation and sequencing were conducted at Macrogen (Kyoto, Japan).

The raw sequencing data have been deposited in the DNA Data Bank of Japan (DDBJ) under accession numbers DRR404790–DRR404793 and DRR308102.

Whole-genome data from other regional populations and subspecies of the leopard cat were obtained from the GenBank database as follows:

- Amur (Korea): SRR3042235;
- Borneo: SRR4426162;
- Malay Peninsula: SRR4426163;
- Amur: SRR4426164;
- Indochina: SRR4426173.

As an outgroup for phylogenetic analysis, we also obtained the whole-genome sequence of the fishing cat (*Prionailurus viverrinus*) from GenBank (SRR4426169, Mishima, Shizuoka, Japan).

### 2.3. Filtering Reads and Variant Calling

Low-quality reads were trimmed using Fastp ver. 1.33 [7]. Nextera adaptor and other Illumina primer sequences were clipped with default settings. A sliding window trimming step removed sequences whose average quality was <30. The filtered reads were mapped to the domestic cat genome (Felis_catus_9.0), wherein repetitive regions were masked (hard mask) and sex chromosomes were removed using BWA ver.0.7.10 [8] with the BWA–MEM algorithm and default settings. SAM files were converted to BAM files, sorted, and duplicate reads removed using Samtools ver. 1.11 [9]. To prepare SNP datasets for downstream analyses, we first generated a BCF file from BAM files using Bcftools ver.1.9 [10] *mpileup* with the options “-C50, -Ou”. The resulting BCF file was then processed using two different variant calling strategies optimized for specific analytical purposes.

For PSMC analysis, consensus FASTQ sequences were generated by calling variants with *bcftools call* “-c”, followed by conversion to FASTQ format using vcfutils.pl *vcf2fq*. Sites with extremely low or high read depths were filtered out based on the average sequencing coverage: positions with a depth below one-third of the average coverage (or below 5 if one-third coverage was less than 5), and those above twice the average coverage, were excluded. The resulting FASTQ file was then compressed using gzip and saved with a “.diploid.fq.gz” extension, following the naming convention required for input files to PSMC. This extension indicates that the file contains diploid consensus sequences in FASTQ format, which PSMC expects as input for inferring historical population demography.

For SMC++ and genetic clustering analyses, variant sites were identified using a multiallelic calling model, and VCF files were compressed in gzip format. After generating individual VCF files for each sample using the multiallelic calling model, we applied quality filtering to exclude low-confidence variants and sites with anomalous sequencing depth. Specifically, each VCF file was filtered using *bcftools filter* with the following criteria: variants with a QUAL score below 20 were labeled as “LowQual”, and sites with a read depth (DP) lower than one-third of the sample’s average coverage (or below 5 if one-third coverage was less than 5), or greater than twice the average coverage, were excluded. This step was performed for all 11 samples individually, using coverage-specific thresholds adjusted per sample. Filtered VCF files from all samples were then merged into a single multi-sample VCF file using *bcftools merge*, with the output in compressed VCF format. This joint genotyping dataset was subsequently used for demographic inference with SMC++ and distance-based genetic clustering (neighbor-joining tree).

### 2.4. Inferring Demographic History with PSMC and SMC++

The consensus diploid data was analyzed using the PSMC software package ver. 0.6.5-r67 (https://github.com/lh3/psmc accessed on 1 July 2021) with “*fq2psmcfa*” option and “-q 20” setting. PSMC modeling was outputted using the following “psmc” options: 25 cycles of the algorithm (-N25), the upper limit of the most recent common ancestor was 15 (-t15), and the initial θ/ρ was 5 (-r5). The PSMC outputs were obtained with 64 atomic time interval parameters (the –p parameter used for PSMC was set to “1 × 4 + 25 × 2 + 1 × 4 + 1 × 6”), indicating that the first *Ne* parameters cover the first four atomic time intervals, each of the next twenty-five parameters cover two atomic intervals, the twenty-seventh parameter covers four intervals, and last parameter covers the last six time intervals [5]. To check the variance of *Ne*, 100 bootstrap was performed with 5 Mb segmented consensus sequences and random resampling by replacement. While scaling the plot of PSMC results, generation time (*g*) and average mutation rate per site per generation (*μ*) were set to 6.44 years [11] and 1.0 × 10^−8^, respectively. The mutation rate was set to 1.0 × 10^−8^ per site per generation, a commonly used estimate for mammals [12,13].

The SMC++ is a population genetics method and software for inferring demographic history through time from genome sequences [14]. We used the SMC++ model to infer the demographic history of the TLC population. Four TLC individuals and one individual from the Korean population were used in the analysis.

To exclude unreliable genomic regions from demographic inference using SMC++, we constructed a genomic mask (mask.bed.gz) based on ambiguous bases in the reference genome, regions with high levels of missing genotype data, and low-complexity sequences. This mask ensured that only high-quality, informative genomic regions were retained for analysis. We first extracted a subset of individuals from the merged multi-sample VCF file, including only the six samples used for SMC++ analysis: four TLC individuals, one Korean individual, and one ILC. This subset VCF was generated using *bcftools view*. Ambiguous regions in the reference genome—stretches of consecutive ‘N’ bases—were identified using *seqtk cutN* in Seqtk ver. 1.3-r106 (https://github.com/lh3/seqtk accessed on 1 July 2021). Only regions ≥ 100 bp were retained, and their coordinates were recorded in BED format for exclusion. In parallel, we assessed genotype missingness using Vcftools ver. 0.1.17, calculating site-level missing data across the six target individuals. Sites where ≥10% of individuals lacked genotype calls were identified and recorded as single-base entries in BED format. These two sets of BED intervals (ambiguous regions and missing data regions) were concatenated, sorted, and merged using *bedtools merge*. The resulting BED file was compressed using bgzip and indexed with tabix, producing the final mask.bed.gz. This mask was used in subsequent SMC++ analyses to restrict inference to reliable genomic regions.

We employed SMC++ (version: terhorst/smcpp:latest) to infer historical effective population sizes for each target population. For each, the filtered VCF was converted to SMC++ input format using *smc++ vcf2smc*, specifying the focal individual, population assignment, and the mask file. Demographic history was then inferred using *smc++ estimate*, with appropriate mutation rate and generation time settings. Finally, demographic trajectories were visualized with *smc++ plot*, showing changes in effective population size over time. For scaling, we applied the same mutation rate (μ) and generation time (g) parameters used in the PSMC analysis.

### 2.5. Analysis of Subspecies Divergence Time

We analyzed the divergence time between the TLC and the Korean leopard cat populations using pseudo-diploid sequences, following the method of Prado-Martinez et al. [15]. These sequences were created by randomly selecting one allele at each site from the haploid genomes of the two populations. In the PSMC plots of pseudo-diploid sequences, the point at which the estimated *Ne* approaches infinity is interpreted as the divergence time between the two populations. Pseudo-diploid sequences were constructed using the Seqtk program by merging the haploid sequences from each population. We used the “-rq20” option to retain nucleotides with a minimum of 20-fold sequencing coverage. The resulting pseudo-diploid sequences were analyzed using PSMC under the same conditions described above.

In addition, we inferred divergence time using the split model implemented in SMC++. This model jointly fits a clean-split demographic history using marginal estimates from each population. First, we generated SMC input files for each population independently using the *smc++ vcf2smc* command, assigning individuals to their respective populations. Marginal demographic histories were inferred with the *smc++ estimate* command. Subsequently, we constructed joint SMC files capturing the joint allele frequency spectrum by including individuals from both populations in the *smc++ vcf2smc* command. These joint SMC files capture shared genealogical signals between the populations and are required for the split model. We then applied the *smc++ split* command, providing the final model JSON files from each marginal estimate along with the joint SMC files. This step refined the marginal models into a joint demographic model and yielded an estimate of the divergence time. The inferred split time and joint demographic trajectories were visualized using *smc++ plot*.

### 2.6. Heterozygosity, Inbreeding Coefficient, and the Genome Difference Rate Among Populations

We performed phylogenetic analysis, inbreeding coefficient estimation, and heterozygosity calculation using SNP data derived from a multiallelic calling model across all individuals.

Variant sites were first filtered using PLINK ver. v1.90b6.21 [16] with the following criteria: SNPs with more than 10% missing genotypes (--geno 0.10) and minor allele frequency below 5% (--maf 0.05) were removed; individuals with more than 10% missing data were excluded using (--mind 0.10). Non-standard chromosome names were allowed (--allow-extra-chr). The filtered dataset was then converted into PLINK binary format (BED).

Pairwise genetic distances were calculated using allele-sharing metrics (allele count-based Hamming-like distance) with PLINK using the “--distance” option. The resulting distance matrix was reformatted to MEGA format and used to construct a genetic clustering with the neighbor-joining (NJ) method implemented in MEGA v12.0.11 [17].

Inbreeding coefficients (*F*) were calculated using the “--het” option in PLINK. We estimated F values separately for 10 leopard cat individuals and 4 TLC individuals, using the “--keep” option to filter the respective individuals for each analysis. The resulting het file was used to derive inbreeding coefficients. To estimate genome-wide heterozygosity (*He*), we utilized diploid consensus sequences (.diploid.fq.gz) generated during the preprocessing stage of PSMC analysis. The “.diploid.fq.gz” files were first converted to FASTA format using the Seqtk. We then analyzed the resulting sequences to quantify homozygous and heterozygous sites. Homozygous positions were defined as uppercase unambiguous bases (A, T, C, G), while heterozygous positions were defined as the six IUPAC ambiguity codes (R, Y, M, K, S, W). All other characters, including lowercase bases and undetermined nucleotides (e.g., N), were excluded. The total number of evaluated positions (*L*) was calculated as the sum of homozygous and heterozygous sites. Genomic heterozygosity (*He*) was then computed as the proportion of heterozygous sites over the total number of evaluated bases, using the following formula:

*H*e = *d*/(*nd* + *d*)

where *d* is the number of heterozygous positions and *nd* is the number of homozygous positions.

## 3. Results

### 3.1. The Result of Sequences

The sequence amounts obtained from four TLC individuals (22,694, 22,697, 22,698, 22,699) and one ILC were 137 Gb, 119 Gb, 118 Gb, 124 Gb, and 207 Gb, respectively, and the coverage obtained was 58x, 50x, 50x, 52x, and 87x, respectively.

### 3.2. Inference of Effective Population Size

**Figure 2:**
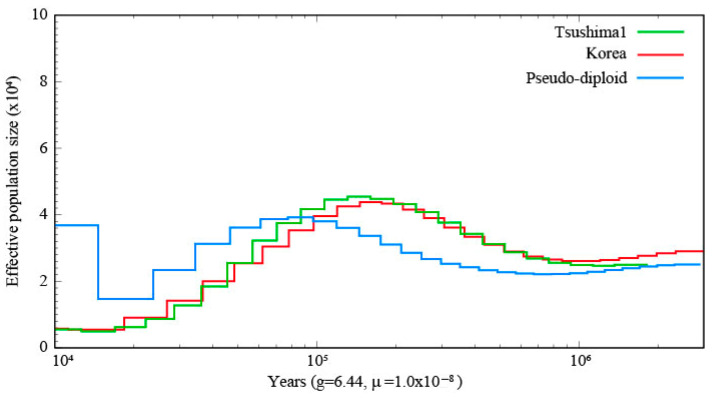
Demographic history of the two leopard cat populations (Tsushima and Korea) and their population divergence time inferred by PSMC analysis. The effective population sizes (*Ne*) of the Tsushima leopard cat (green) and the Korean population (red) were estimated using PSMC analysis. The population divergence was assessed using pseudo-diploid sequences, with the resulting PSMC curve (blue) approaching infinity at the inferred divergence point between the two populations. All trajectories were scaled using a generation time of 6.44 years and a mutation rate of 1.0 × 10^−8^ per site per generation.

**Figure 3:**
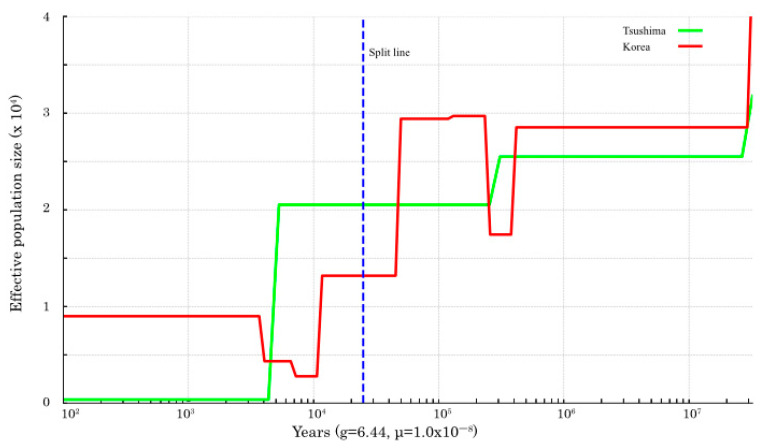
Demographic history of the two leopard cat populations (Tsushima and Korea) inferred by SMC++ analysis. The effective population sizes (*Ne*) of the Tsushima leopard cat (green) and the Korean population (red) were estimated based on whole-genome data. The blue dashed line represents the estimated divergence time between the two populations. Note that some estimates may be influenced by genome coverage falling below the recommended 20-fold threshold for reliable inference using the PSMC method. All trajectories were scaled using a generation time of 6.44 years and a mutation rate of 1.0 × 10^−8^ per site per generation.

The PSMC results of the one TLC individual and the Korean population are shown in Figure 2, and the PSMC plot including four TLC individuals and the Korean population is shown in Supplementary Figures S1 and S2. The demographic history of the TLC and the Korean population showed similar trends. *Ne* size of both populations increased until about 100,000 years ago and then decreased until about 10,000 years ago. In the SMC++ plot, throughout the analyzed period, the TLC population exhibited a sustained decline in effective population size, with marked reductions occurring approximately between 30,000 and 20,000 years ago and 5000 and 4000 years ago (Figure 3). The PSMC for leopard cats in other regions is shown in Supplementary Figure S3. The ILC showed the lowest *Ne* throughout the entire time series compared to other regions. The SMC++ analysis revealed that ILC has maintained a consistently low *Ne* throughout its evolutionary history (Supplementary Figure S4).

### 3.3. Inference of Divergence Time Between the TLC and Korean Population

The *Ne* size, based on the pseudo-diploid sequences, increased from 20,000 to 1000 years ago, but did not increase indefinitely (Figure 2 and Supplementary Figure S2). According to SMC++ inference, the divergence between the two populations occurred approximately 30,000 to 20,000 years ago (Figure 3).

### 3.4. Heterozygosity, Inbreeding Coefficient, and Genetic Distances Among Populations

A total of 12,505,692 single-nucleotide polymorphisms (SNPs) passed the filtering criteria across all populations, including the fishing cat. When restricted to leopard cat populations only (excluding the fishing cat), 9,726,075 variants remained after filtering.

The heterozygosity and inbreeding coefficient for each population are shown in Table 1. The TLC had a low heterozygosity rate, less than half that of the Korean population. The ILC had a very low heterozygosity (0.000025). Although the other four populations (Borneo, Malay Peninsula, Amur, and Indochina) are included only for reference because of their low coverage—thereby limiting the reliability of heterozygosity estimates and requiring that the results be interpreted with caution—three of these populations showed similar heterozygosity levels (0.001820–0.002129), whereas the Amur population exhibited a notably lower value (0.000808). The inbreeding coefficient of the TLC was higher than that of the Korean population, while that for the ILC was extremely high. The inbreeding coefficients of individuals within the TLC population using 3,114,908 SNPs that passed the filtering step ranged from 0.01834 to 0.025.

**Figure 4:**
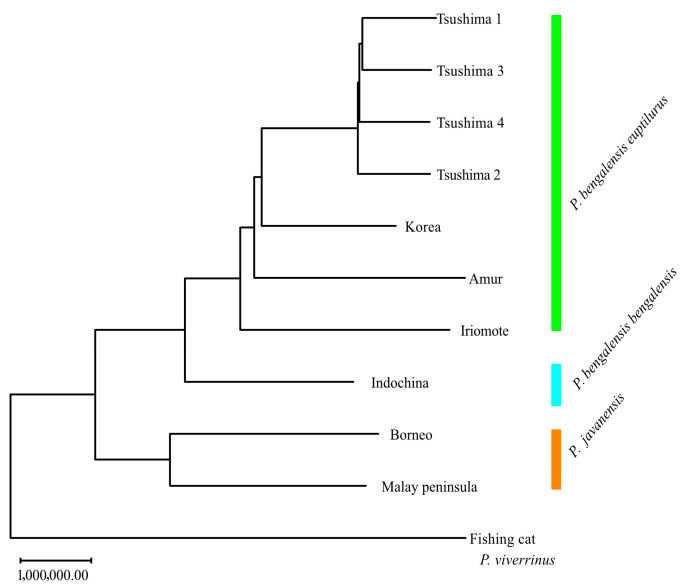
Neighbor-joining tree of leopard cat species/subspecies based on allele count distances from 12 M SNPs. The tree was constructed using the neighbor-joining algorithm from a distance matrix derived via PLINK, based on allele mismatches across genome-wide SNPs. Branch lengths represent relative genetic divergence among species/subspecies. The Tsushima leopard cat clustered most closely with the Korean population, followed by Amur and Iriomote populations.

Genetic distance and NJ trees based on them are shown in Supplementary Table S1 and Figure 4, respectively. The TLC was most closely related to the Korean population, followed by the Amur and the ILC. The leopard cat was divided into two groups: one comprising the TLC, Korea, Amur, ILC, and Indochina populations, and the other comprising the Borneo and Malay Peninsula populations.

## 4. Discussion

Understanding the demographic history and genetic diversity of the TLC is critical for developing effective conservation strategies for this insular population. Our results clarify the historical population dynamics and genetic status of the TLC, while also providing preliminary insights into the ILC. Although the TLC is inferred to have diverged from the continental population approximately 100,000 years ago [18], the PSMC showed that the population dynamics of the TLC and the Korean populations have remained similar after the divergence. Since the last glacial period occurred between 115,000 and 11,700 years ago, and the demographic decline (100,000 to 10,000 years ago) coincided with this time, the population size probably declined due to the cold climate during this period. The population demographics of the four individuals in the SMC++ analysis were similar to those in the PSMC analysis; moreover, unlike PSMC, SMC++ allowed us to estimate the population demographics for the period from 10,000 years ago to more recent years. The population size decreased about 5000 years ago. SMC-based methods have limitations in resolving recent demographic events [19]. The most recent population size that can be estimated by these methods depends on the time to the most recent coalescence event that occurred in the sample, and, thus, becomes out of date if the sample size is small [19]. In the case of the TLC, population estimates were limited to 10,000 years ago by PSMC. SMC++ has advantages over PSMC, particularly in its ability to infer recent changes in effective population size over the past several thousand years with higher resolution. However, the performance of SMC++ is dependent on the number of individuals analyzed; thus, incorporating a larger number of individuals is desirable to improve the robustness of demographic inferences. Increasing the number of analyzed individuals and incorporating complementary methods, such as Site Frequency Spectrum-based approaches (e.g., FASTSIMCOAL2 [20]) or identity-by-descent-based methods (e.g., IBDNe [21]), will enable a more comprehensive reconstruction of population history across different time scales.

Divergence time estimates based on pseudo-diploid analysis suggest that complete separation between the TLC and continental (Korean) populations had not occurred (Figure 2), although divergence likely began 30,000–20,000 years ago as inferred by SMC++ (Figure 3). These estimates are more recent than those from mitochondrial DNA (100,000 years ago) [18]. Since the Tsushima island and the mainland are thought to have been separated by a 10–20 km wide strait during the Last Glacial Maximum [22], the leopard cat being a frequent migrant seems unlikely. However, dispersal events like those of the Japanese wolf (*Canis lupus hodophilax*), which migrated from the continent to Japan between 57 and 35 ka and 37 and 14 ka [23], suggest that occasional crossings were possible. Collectively, these findings underline the need to investigate post-glacial gene flow in greater detail.

The heterozygosity rate in the TLC was lower than in any other regional population, except the ILC. This value was lower than that for endangered species such as the giant panda and the chimpanzee, and close to that for the Bornean orangutan and the eastern gorilla [24,25,26,27,28,29]. High inbreeding coefficients (up to 0.25) in some individuals suggest that inbreeding is ongoing and requires careful monitoring. Although only a single ILC individual was analyzed, it exhibited the lowest heterozygosity among all populations studied, including previous reports [28]. Its long-term isolation, following divergence from the mainland 200,000–180,000 years ago, may explain its significantly reduced diversity. The SMC++ analysis also indicated a persistently low *Ne*, likely due to founder effects and restricted gene flow. While these results must be interpreted cautiously due to the limited sample size, they emphasize the urgency of expanding genetic sampling for the ILC. The preliminary evidence suggests that the ILC may face an even greater conservation risk than the TLC, reinforcing the need for population-specific management and genetic monitoring.

The neighbor-joining tree showed that the leopard cat was divided into two major clusters: one group including the TLC, Korean, Amur, ILC, and Indochina populations, and another group including the Borneo and Malay Peninsula populations. Based on whole mitochondrial analysis [30], the leopard cat is divided into two groups—the northern (Mainland) and the southern (Sunda)—and the results of our whole-genome analysis are also consistent with this classification. The mainland lineage, which includes the TLC and the ILC, is divided into two subspecies: the Bengal leopard cat (*P. b. bengalensis*) and the Amur leopard cat (*P. b. euptilura*). While the taxonomic status of the ILC remains debated, its genetic clustering supports its recognition as a separate conservation unit. Further analyses involving more continental individuals will help clarify gene flow and divergence between insular and mainland populations.

Together, these genomic insights provide a foundation for defining appropriate conservation units and implementing informed, population-specific strategies—such as translocation, assisted gene flow, or ex situ breeding—to mitigate further genetic erosion.

## 5. Conclusions

This study explores the demographic history and genetic divergence of the endangered TLC through whole-genome sequencing and coalescent-based analyses. The TLC population exhibits a sustained decline in effective population size, with divergence from the Korean population estimated between approximately 30,000 and 20,000 years ago. Genetic analyses revealed extremely low levels of heterozygosity alongside high inbreeding coefficients, suggesting limited adaptive potential and posing serious conservation concerns. Phylogenetic inference positioned the TLC as the closest relative to the Korean population, forming a distinct northern clade. Notably, our findings also offer preliminary insights into the critically low genetic diversity of the ILC, although these observations are based on a single specimen.

These results have significant implications for conservation strategies targeting the TLC. The combination of low genetic diversity and persistent inbreeding underscores the need for long-term genetic monitoring to mitigate further erosion of genetic variation. Demographic reconstructions revealed historical bottlenecks likely influenced by climatic shifts or anthropogenic pressures, providing a temporal framework for assessing future risks. Furthermore, population clustering analyses reinforce the imperative to preserve the genetic distinctiveness of the TLC—an essential consideration in translocation and captive breeding initiatives.

In conclusion, these genomic findings establish a solid foundation for defining evolutionarily significant conservation units and prioritizing management strategies tailored to the unique evolutionary trajectory of the Tsushima leopard cat. Future research incorporating broader genomic sampling and complementary demographic approaches will be essential to refine these insights and enhance evidence-based conservation planning.

## Figures and Tables

**Table 1:** Heterozygosity and inbreeding coefficient in leopard cat population.

	Heterozygosity	Inbreeding Coefficient Among Leopard Cats	Inbreeding Coefficient Among Tsushima Leopard Cats
Tsushima leopard cat			
Tsushima 1	0.000635	0.6926	0.1834
Tsushima 2	0.000641	0.6931	0.1897
Tsushima 3	0.000576	0.7149	0.25
Tsushima 4	0.000620	0.6971	0.2015
Korea	0.001545	0.4011	-
Iriomote	0.000025	0.9276	-
Borneo	0.001820	0.5481	-
Malay Peninsula	0.002129	0.4477	-
Amur	0.000808	0.6926	-
Indochina	0.001871	0.4543	-

## Data Availability

The raw sequencing data have been deposited in the DDBJ Sequence Read Archive under the accession numbers DRR404790–DRR404793 and DRR308102.

## Supplementary Materials

The following supporting information can be downloaded at: https://www.mdpi.com/article/10.3390/biology14070880/s1, Supplementary Figure S1: demographic history of the Tsushima leopard cat by PSMC analysis; Figure S2: demographic history of the Tsushima leopard cat, Korean population and pseudo-diploid between Tsushima and Korea population by PSMC analysis with 100boostrap; Figure S3: demographic histories of five regional populations of the leopard cat by PSMC; Figure S4: demographic histories of the Iriomote leopard cat by SMC++; Table S1: pairwise genetic distance matrix based on 12M SNPs.

## Abbreviations

The following abbreviations are used in this manuscript:

PSMC	pairwise sequentially Markov coalescent
*Ne*	effective population size
