# Supplementary material for: Assessing the Population Demographic History of the Tsushima Leopard Cat and Its Genetic Divergence Time from Continental Populations
Source: Biology (Basel). 2025 Jul 18;14(7):880. doi: 10.3390/biology14070880 (PMC12292410; doi:10.3390/biology14070880)
Supplement: Supplementary file 1 [file biology-14-00880-s001.zip › biology-3750240-supplementary.pdf]

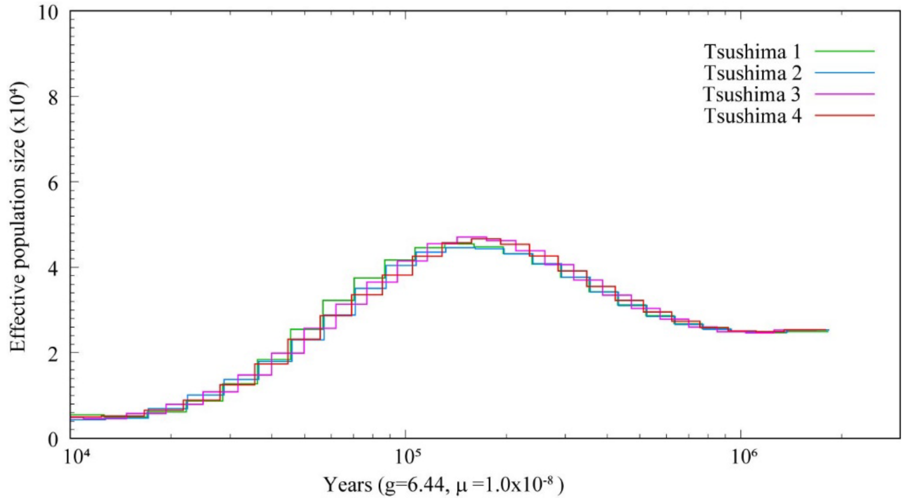

Supplementary Fig. 1 Demographic history of the Tsushima leopard cat by PSMC analysis.

The PSMC results for the four individuals were similar, and Fig. 1 was considered representative of the PSMC results for the Tsushima leopard cat.

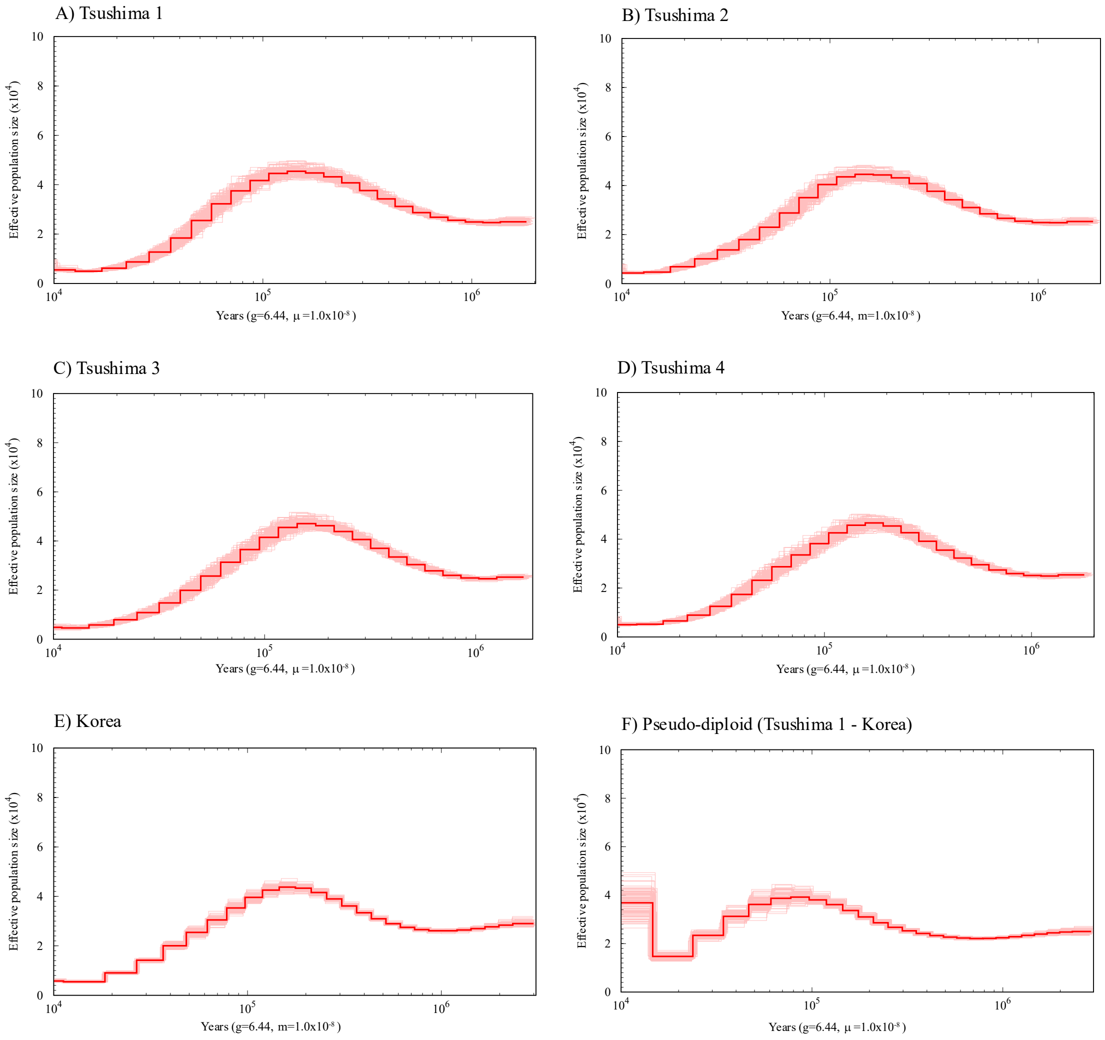

Supplementary Fig. 2 Demographic history of the Tsushima leopard cat, Korean population and pseudo-diploid between Tsushima and Korea population by PSMC analysis with 100boostrap. A) Tsushima 1, B) Tsushima 2, C) Tsushima3, D) Tsushima 4, E) Korea, F) Pseudo-diploid (Tsushima 1 – Korea). Darker lines represent the population size inference, and lighter, thinner lines represent 100 bootstraps replicates to quantify uncertainty of the inference.

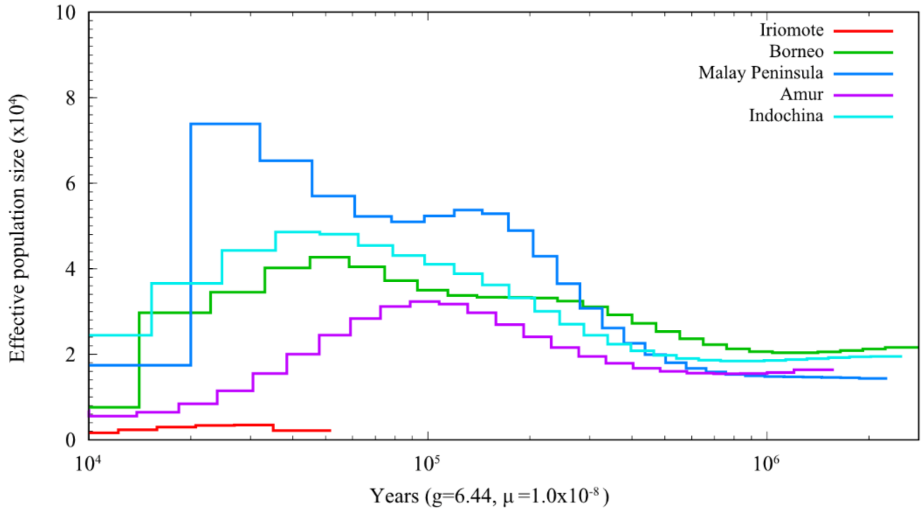

Supplementary Fig. 3 Demographic histories of five regional populations of the leopard cat by PSMC. Four out of the five populations (Borneo, Malay Peninsula, Amur, and Indochina) had genome coverage below the recommended 20-fold threshold for reliable inference by the PSMC method. These results should therefore be interpreted with caution due to potential biases introduced by low sequencing depth.

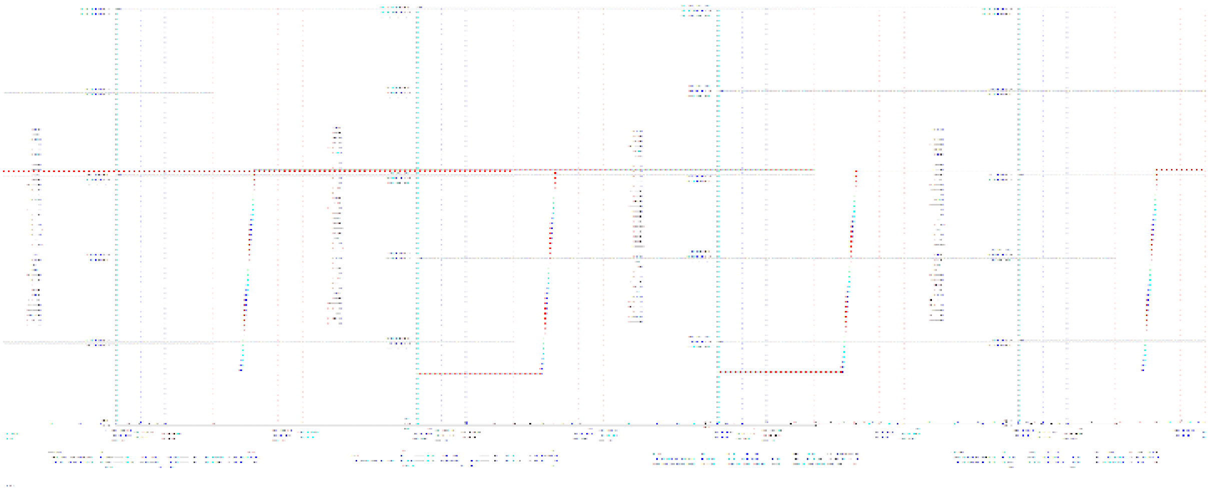

Supplementary Fig. 4 Demographic histories of the Iriomote leopard cat by SMC++.

Supplementary Table 1 Pairwise Genetic Distance Matrix Based on 12M SNPs

	Tsushima 1	Tsushima 2	Tsushima 3	Tsushima 4	Korea	Iriomote	Borneo	Malay peninsula	Amur	Indochina
Tsushima 2	2182970									
Tsushima 3	2011390	2012720								
Tsushima 4	2076720	2023200	2004600							
Korea	4291680	4262640	4317980	4268550						
Iriomote	5551750	5520940	5558280	5528690	5356820					
Borneo	8897630	8886630	8912920	8887500	8195660	9004310				
Malay peninsula	8635950	8623980	8655190	8634970	8127430	8830360	5705110			
Amur	5482780	5471290	5505470	5479710	4941780	6381620	9053430	8498000		
Indochina	5959920	5948660	5988640	5950120	5096440	6169420	7575850	7342300	6143530	
Fishing cat	12448000	12435900	12458000	12436400	11849900	12515900	11272000	11774700	12559500	11359200

This table presents allele count–based genetic distances between individuals, calculated using PLINK across 12 million (12,505,692) SNPs shared among all samples, including the outgroup. Values indicate the total number of differing alleles per pair.
